# Supplementary material for: HTR1A, TPH2, and 5-HTTLPR Polymorphisms and Their Impact on the Severity of Depressive Symptoms and on the Concentration of Tryptophan Catabolites during Hepatitis C Treatment with Pegylated Interferon-α2a and Oral Ribavirin (PEG-IFN-α2a/RBV)
Source: Cells. 2023 Mar 22;12(6):970. doi: 10.3390/cells12060970 (PMC10046909; doi:10.3390/cells12060970)

Figure S1. Chromatographic analysis of aqueous standard solution (A: KYN at 5.2 min and TRP at 12.6 min; C: KYNA at 6.8 min, TRP at 7.5 min, and AA at 9.4 min) and typical human serum (B: KYN and TRP; D: KYNA, TRP, and AA).

A

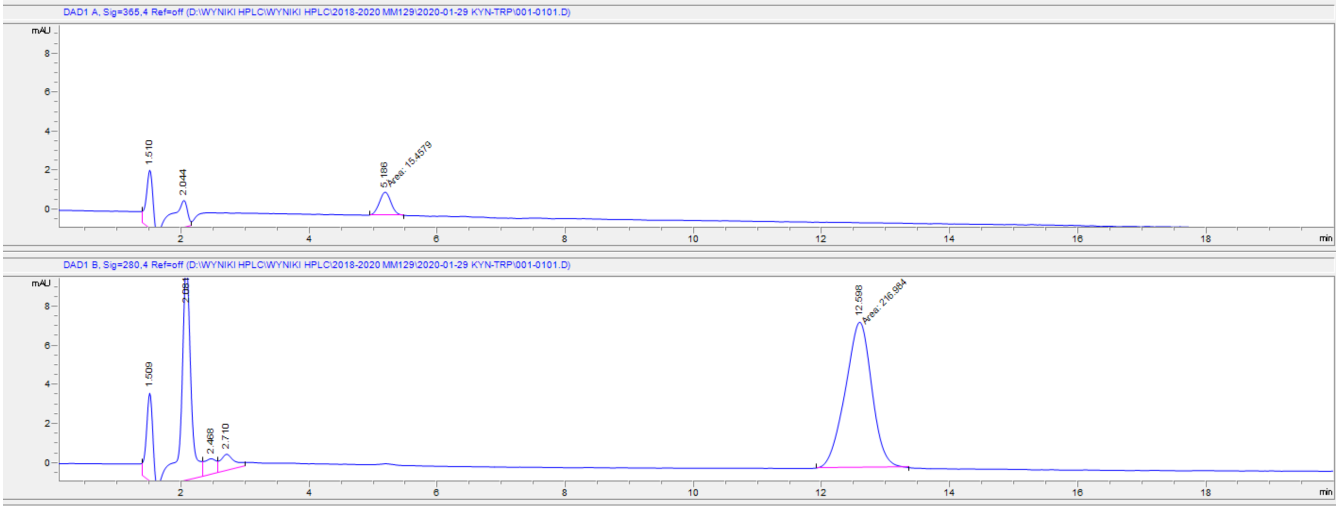

B

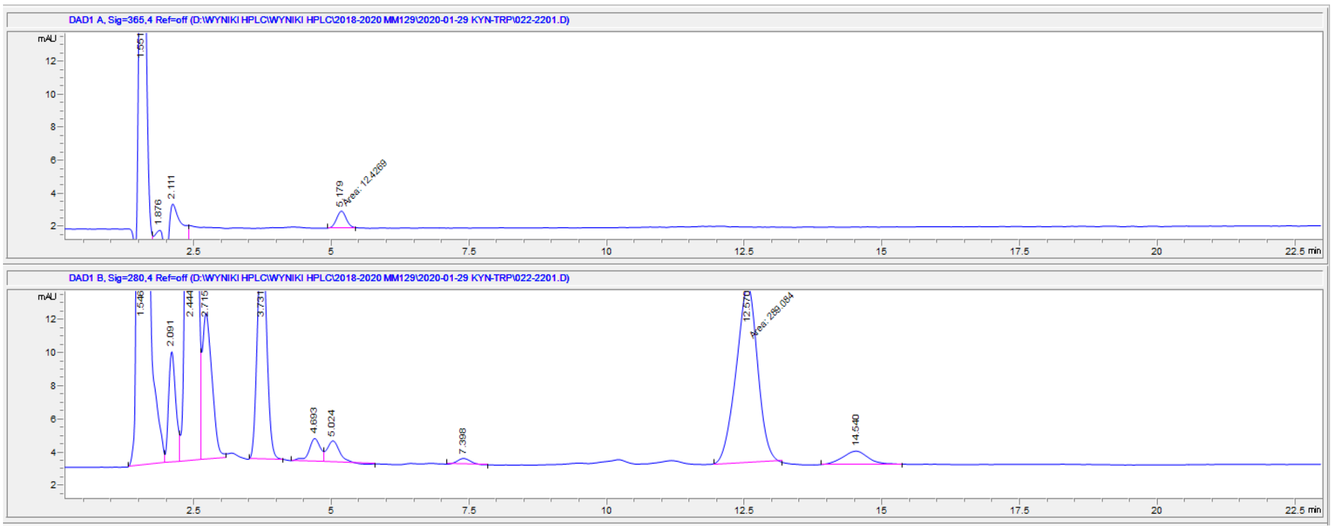

C

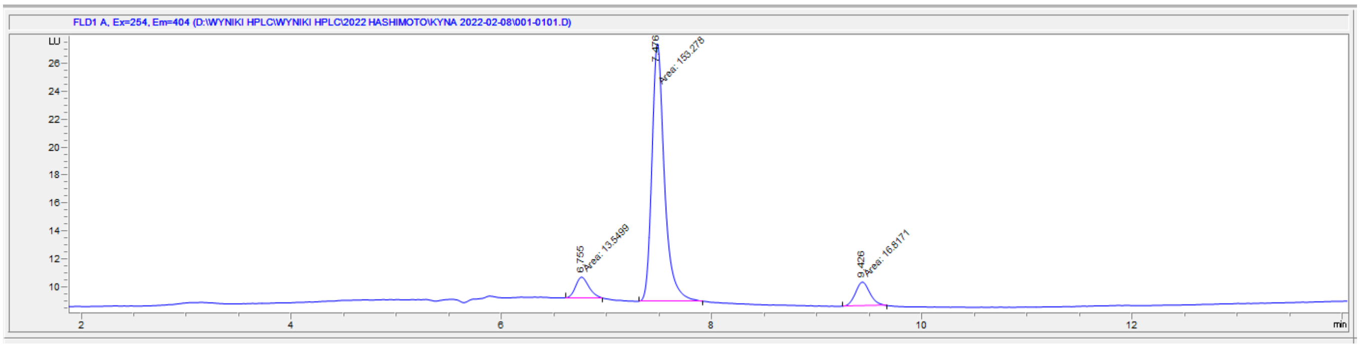

D

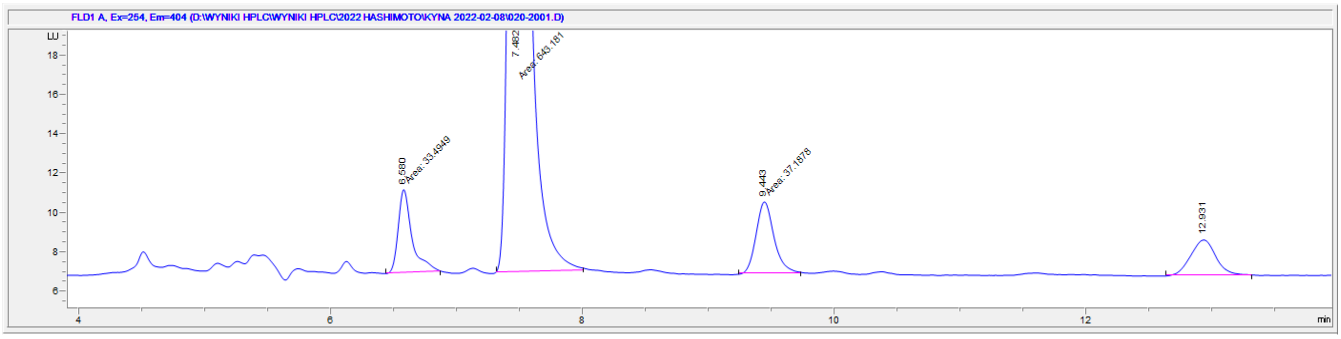

Supplement: Supplementary file 1 [file cells-12-00970-s001.zip › cells-2205203-supplementary.pdf]
